# Localization of Basicranium Midline by Submentovertex Projection for the Evaluation of Condylar Asymmetry

**DOI:** 10.1155/2012/285693

**Published:** 2012-01-18

**Authors:** Michele Maglione, Fulvia Costantinides

**Affiliations:** Unit of Oral Surgery, Department of Medical and Surgical Sciences, Dental School, University of Trieste, Piazza dell'Ospitale 1, 34127 Trieste, Italy

## Abstract

The purpose of this research was to compare the reliability of two different methods for cranial midline localization through cephalometric analysis of mandibular condyle asymmetries. A retrospective cohort study was performed analyzing consecutively the SMV radiograms of 47 patients undergoing oral surgery before orthodontic treatment at the Dental School, University of Trieste (Italy) from 2003 to 2008. Two different cephalometric analyses were used to identify the basicranium midline (Tracing 1: initial landmarks = craniostat ear rods; Tracing 2: initial landmarks = spinosum foramina), and the left/right symmetry ratio (SR) for four parameters (condylar length, condylar angle, intra-condylar hemidistance, extra-condylar hemidistance) was calculated. The main result showed that no significant statistical difference between the SRs of the intra-condylar and extra-condylar hemidistance obtained with the same tracing was found (*t*-test; *P* = NS; C.I. 95%). Conversely, the difference between the SRs obtained with the two different tracings was statistically significant (*t*-test; *P* < 0.000; C.I. 95%). In conclusion, if the analysis of condylar asymmetries is performed in growing subjects, utilization of anatomic references such as the neurovascular foramina seems to guarantee a lower error compared to non-fixed references such as ear rods.

## 1. Introduction

Without considering major facial deformities typically associated with various syndromes, a small degree of craniofacial asymmetry is always present in all subjects with normal biometric parameters, although this asymmetry is rarely appreciable and is often unnoticed [1].

Craniofacial asymmetry is often a cause of major diagnostic difficulties in orthodontics. Diagnosis of asymmetry and of its localization could be essential for planning treatment and evaluating the results of orthognathodontics or maxillofacial surgery [2, 3].

Asymmetry of the craniofacial complex can be evaluated only with appropriate radiological projections and cephalometric measurements on posteroanterior and submentovertex (SMV) radiograms or by using three-dimensional (3D) computed tomography (CT) imaging [4–7].

Furthermore, prior to combined orthodontic/orthognatic surgery, radiological images are recommended to evaluate if there were preexisting temporomandibular pathologies in the patients to identify or prevent temporomandibular disorders that could heavily influence the postsurgical function. Altered anatomical condylar position and bone degeneration (osteoarthrosis) are often associated with the angle jaw discrepancies/malocclusion [8]. Those condylar alterations have to be detected and quantified to optimize the pretreatment diagnosis or post-treatment follow-up. This represents a necessary approach also in children that often display functional temporomandibular disorders [9].

Although magnetic resonance represents the “gold standard” in TMD diagnosis [10], the SMV radiogram gives at the same time an immediate localization and quantification of bone condylar asymmetries and the visualization of other facial and mandibular structures that could be involved in the craniofacial asymmetry.

Ritucci and Burstone and Nahoum et al. [11, 12] underlined the importance of the SMV view in analyzing craniofacial asymmetries, in particular because other radiograms, such as lateral projections, cannot detect asymmetries in the sagittal and coronal planes.

The system of cephalometric coordinates to evaluate the symmetry of bone structures of splanchno- and neurocranium takes as its main reference the sagittal symmetry axis, whose identification has been examined by numerous authors over the last thirty years [13–18].

Cheney proposed a midsagittal plane passing through the nasion and the anterior nasal spine considering that this plane crosses the prosthion and the menton in subjects with a symmetrical face [13]. Conversely, in a cephalometric study in a posteroanterior cranial projection, Sutton found that the anterior nasal spine, the prosthion and the menton are rarely aligned on the same line, confuting the validity of these points for tracing the symmetry axis [14]. Berger proposed examining asymmetries in the cranial basilar projection by tracing the midline passing through the vomer, the posterior portion of nasal septum and the crista galli process [15]. Marmary et al. identified the craniofacial midline as the line perpendicular to the midpoint between the right and the left spinosum foramina in the basilar view, as they believed it remained constant during cranial development [17]. The same method is used by Williamson et al. [18].

More recently, in a 3D CT study, Katsumata et al. [6] selected the midsagittal reference plane passing through points S, N and Dent, whereas Uysal and Malkoc [4] and Janson et al. [5] use the transporionic axis to trace, orthogonally, the midsagittal axis considering that the line connecting the midpoint of the external acoustic meatuses (or Mei) is superimposable to the line connecting the tip of ear rods on SMV cephalograms.

Although the key to evaluating asymmetries is defining the criteria to determine the cranial midline, the existence and utilization of different procedures to identify the ideal midline underlines that no clear, universally accepted, method currently exists for the evaluation of craniofacial asymmetries. Furthermore, until now no study exists evaluating the comparison between the utilization of ear rods and spinosum foramina as starting points for drawing basicranium midline on SMV radiograms.

The aim of the study was to compare the validity and reliability of two different methods for localization of the cranial midline through a cephalometric analysis of asymmetries of the mandibular condyles in a representative adult population.

The hypothesis that the spinosum foramina represent the most reliable starting points for tracing the coordinate system to identify transverse craniofacial asymmetries on SMV radiograms, was tested.

## 2. Materials and Methods

This retrospective cohort study was performed by analyzing the SMV radiograms of 47 patients (26 females and 21 males; age range: 21–56 years; mean age 27 ± 14.2 years), all Italians and with Italian parents, who were consecutively selected from orthodontic patients treated at the Dental Clinic of the University of Trieste between 2003 and 2008. All patients needed third molar extraction or mesiodens removal and subsequent planning of orthodontic treatment. Patients met the following criteria: normal growth, normal development, no clinically apparent facial asymmetry, all teeth present (or physiologic denture for age), no second or third class malocclusion, no functional mandibular deviation, no previous orthodontic treatment, no systemic pathologies or syndromes. Furthermore, we analyzed the SMV radiograms of 5 subjects (range age: 4–25 years; mean age 12 ± 9.1 years), 3 of them still during the active growth phase, performed before and after orthodontic therapy, for a total of 10 radiograms. A written informed consensus was obtained by patients or their parents before treatment through a protocol approved by the University of Trieste, Italy. The principles outlined in the Declaration of Helsinki were followed.

### 2.1. Radiographic Technique and Cephalometric Tracings

**Figure 1:**
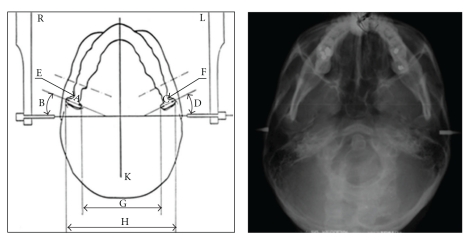
Scheme of the submentovertex radiograms and corresponding digital acquisition. (A) right transverse condylar width, (B) right condylar horizontal angle, (C) left transverse condylar width, (D) left condylar horizontal angle, (E) neck of the right condyle, (F) neck of the left condyle, (G) intracondylar distance, (H) extracondylar distance, (K) midline (orthogonal to the transporionic axis).

Cranial radiographs in SMV projection were obtained by the same operator (M.M.) with an Axial Tome EX II unit (Axial Tome Corporation, San Carlos, CA, USA). The choice of a single operator responded to the need to reduce inter-operator bias during the positioning of the patient and the insertion of the ear rods. Radiograms were performed with the following technique: ear rods were positioned and each patient was asked to rotate the head posteriorly until the Frankfurt plane became parallel to the film cassette [19]. This position was fixed with the aid of the craniostat to allow for reproducibility in the assessment of cranial structure in the horizontal plane [4]. The patient was asked to occlude in centric occlusion under light pressure during exposure. The radiograms were then scanned using a PowerLook 1000 scanner (UMAX Systems GmbH, Willich, Germany) (Figure 1). All cephalometric lines and angles were traced and measured with Microsoft Image Pro plus 5.0 software (Media Cybernetics Inc., Bethesda, MD, USA) after appropriate calibration.

**Figure 2:**
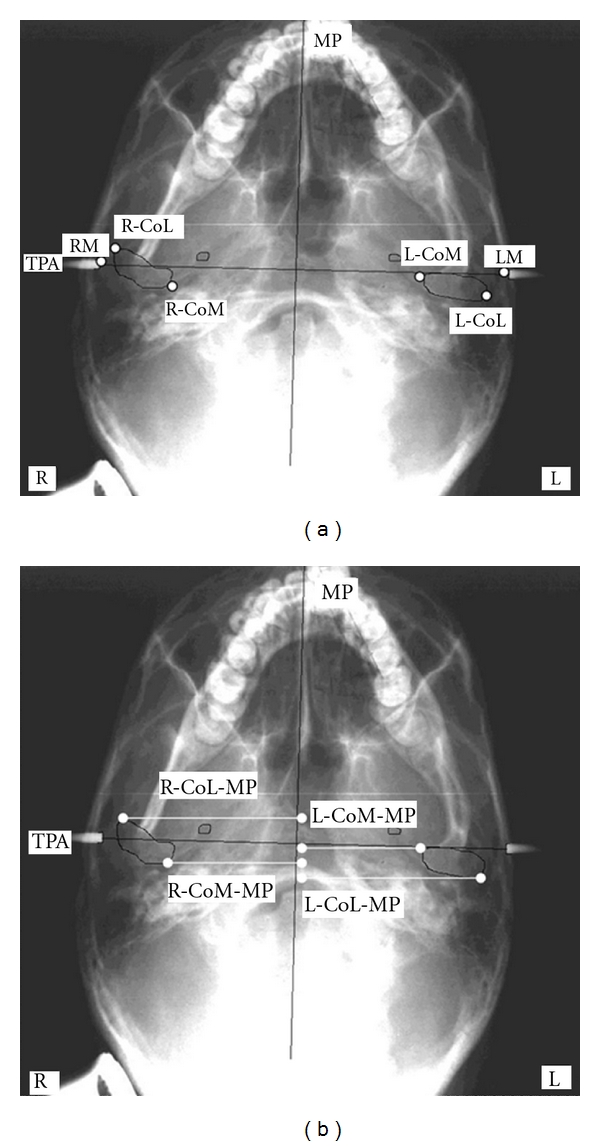
(a-b) Anatomic landmarks and reference planes used in submentovertex cephalometric analysis (Tracing 1). TPA, transporionic axis: line passing through the left and right tip of ear rods corresponding to the line passing through the midpoint of external auditory meatus (LM, left Mei; RM, right Mei); MP, midsagittal axis: perpendicular bisecting TPA; RCoL, right condylion lateralis: most lateral aspect of right condyle; RCoM, condylion medialis: most medial aspect of right condyle; LCoL, left condylion lateralis: most lateral aspect of left condyle; LCoM, left condylion medialis: most medial aspect of left condyle; RCoL-MP, right condylion lateralis-midline: distance from right L-point to MP; RCoM-MP: right condylion medialis-midline: distance from right M-point to MP; LCoL-MP, left condylion lateralis-midline: distance from left L-point to MP; LCoM-MP, left condylion medialis-midline: distance from left M-point to MP.

**Figure 3:**
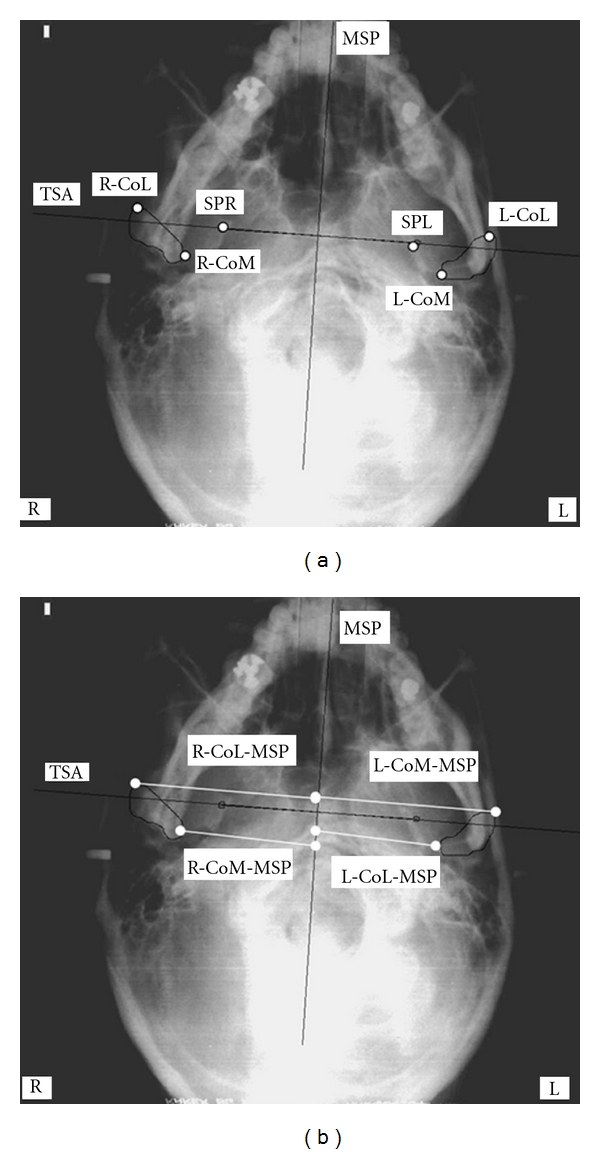
(a-b) Anatomic landmarks and reference planes used in submentovertex cephalometric analysis (Tracing 2). SPR, right foramen spinosum: geometric centre of right foramen spinosum; SPL, left foramen spinosum: geometric centre of left foramen spinosum; TSA, transspinosum axis: line passing through the geometric centre of right and left spinosum points; MSP, midsagittal axis: perpendicular bisecting TSA; RCoL, right condylion lateralis: most lateral aspect of right condyle; RCoM, condylion medialis: most medial aspect of right condyle; LCoL, left condylion lateralis: most lateral aspect of left condyle; LCoM, left condylion medialis: most medial aspect of left condyle; RCoL-MSP, right condylion lateralis-midline: distance from right L-point to MSP; RCoM-MSP: right condylion medialis-midline: distance from right M-point to MSP; LCoL-MSP, left condylion lateralis-midline: distance from left L-point to MSP; LCoM-MSP, left condylion medialis-midline: distance from left M-point to MSP.

The anatomic landmarks used in this study were extrapolated from the SMV analysis developed by Lew and Tay [20] and were: entire outline of the condylar head and identification of internal and external poles, tips of the ear rods and spinosum foramina (Figures 2(a) and 3(a)).

 A single operator (F.C.) performed a digital tracing of each radiogram for five times with the method described below. The resulting mean values of lengths and angles were calculated and considered for statistical analysis to reduce the measurement error.

### 2.2. Midline Localization

Two different cephalometric analyses were chosen to trace the basicranium midline. The first (Tracing 1) considers the craniostat ear rods as initial landmarks. The straight line connecting the tip of the left and right ear rods passes through the left and right midpoint (or left and right mei—LM and RM) of the external acustic meatus (transporionic axis, TPA) [5]; the midline (MP) was established by tracing a perpendicular line crossing the midpoint of the TPA. The second analysis (Tracing 2) uses the spinosum foramina (SF) as main landmarks to identify the axis of symmetry [2]. The outlines of the SF were identified on the radiograms, and the straight line passing through the centers of the left and right SF (points SPL and SPR, resp.) was traced (transspinosum axis, TSA); a second line that passes through the midpoint of the TSA was considered to be the cranial midline (MSP).

### 2.3. Quantification of Condylar Asymmetry

The outlines of the mandibular condyles were traced on each radiogram, and the medial and lateral poles were identified (RCoL, right condylion lateralis: most lateral aspect of right condyle; RCoM, condylion medialis: most medial aspect of right condyle; LCoL, left condylion lateralis: most lateral aspect of left condyle; LCoM, left condylion medialis: most medial aspect of left condyle).

Quantification of the condylar asymmetry was performed using eight parameters:

1. left and right condylar width,
2. left and right condylar angle (the horizontal condylar angle is the angle formed by the straight line passing through the condylar poles and the straight line perpendicular to the midline) [18],
3. intracondylar hemidistance defined as the distance from LCoM and RCoM to MP (Tracing 1) or MSP (Tracing 2),
4. extracondylar hemidistance defined as distance from LCoL and RCoL to MP (Tracing 1) or MSP (Tracing 2).

The anatomic landmarks and reference planes used are shown in Figures 2(a) and 2(b) and 3(a) and 3(b).

On each radiogram, the left/right symmetry ratio (SR) was calculated for Tracing 1 and Tracing 2 with this simple formula:

(1) $$\text{SR}=\;\frac{\text{Left}\;\text{parameter}}{\text{Right}\;\text{parameter}}.$$

The left-side measurement was used as a reference. A SR > 1 indicates that the left side is larger than the right side. A SR < 1 suggests that the right side is greater than the left. A SR = 1 indicates perfect symmetry.

All statistical analyses were performed with the SPSS software package (Statistical Package for Social Sciences, Windows 98, version 10.0, SPSS, Chicago, Ill) using the Student's *t*-test for independent samples.

## 3. Results

Table 1 summarizes the mean values obtained after measurement of the condylar parameters and the SR for Tracing 1 and Tracing 2.

The mean values of the condylar widths and angulations fell within the physiological range [21]. Furthermore, there was substantial equivalence of SR related to the horizontal condylar width and angulation calculated for Tracing 1 and Tracing 2 (*t*-test; *P* = NS; C.I. 95%).

SRs were 1.06 ± 0.11 and 0.99 ± 0.08, respectively, for the distances LCoM and RCoM to MP (Tracing 1) and MSP (Tracing 2) and 1.04 ± 0.09 and 0.98 ± 0.07, respectively, for the distances LCoL and RCoL to MP (Tracing 1) and to MSP (Tracing 2).

Statistical analysis did not reveal any significant difference in the comparison of the SRs of the intracondylar and extracondylar hemidistance using the same tracing (Tracing 1: *t*-test; *P* < 0.2 NS; C.I. 95%; Tracing 2: *t*-test; *P* < 0.9 NS; C.I. 95%). Conversely, the comparison between the SRs obtained using the two different tracings and regarding the same parameter was statistically relevant (intracondylar hemidistance SR for Tracing 1 versus Tracing 2: *t*-test; *P* < 0.000; C.I. 95%; extracondylar hemidistance SR for Tracing 1 versus Tracing 2: *t*-test; *P* < 0.000; C.I. 95%) (Table 2).

Table 3 reports the measurements of the distances between the ear rods and the spinosum foramina for the five control cases. An increment of the distance between the ear rods is appreciable in all three patients in the active growth phase whereas this measure becomes stable in the adult subjects. In all five subjects, the distance between the spinosum foramina remained unvaried.

## 4. Discussion

As reported by Haraguchi et al. [22], nonpathologic facial asymmetry (normal asymmetry), defined as the difference in size between the left and right hemifaces, is relatively common. Asymmetry is often not easily appreciable clinically and could be considered a desirable condition of the craniofacial structures because we perceive it as esthetically pleasing. However during orthodontic planning, the identification and quantification of the asymmetry could be important in patients with clinically significant asymmetry or with pathologic conditions associated with asymmetry [1].

In this context, the SMV radiographic technique represents a useful method to examine the cranial base and to evaluate the rate of asymmetry of the anatomic structures in the axial plane [4]. This kind of projection is more useful than panoramic and posterior/anterior radiography to determine the mediosagittal axis thanks to the excellent visualization of the cranial base structures [23]. However, it should be remembered that radiological techniques, such as cephalometry, could be affected by image size distortion and that their quality and accuracy depend on many variables [18]. Furthermore, cephalometry is bidimensional [24]. Although new tridimensional radiographic techniques have been recently introduced (Cone-beam computed tomography) changing the potential in presurgical diagnosis and pretreatment planning, SMV radiography remains a good choice in clinical practice for the diagnosis of uncomplicated malocclusions, thanks to the ease of execution, the low radiation dose, and the good spatial resolution [25–28].

The analysis of asymmetries requires that all anatomic parameters have to be compared to a symmetry axis (or midline) that is established using stable anatomic references. Williamson et al. [18] underlined the importance of correct determination of landmarks and reliability of measurements to properly interpret the data and apply them to research or clinical practice. Similarly, Trpkova et al. [29] remarked on the need to test the validity of reference lines in evaluating facial asymmetries. They studied how to assess the best sagittal midline from posteroanterior cephalograms and found that a different midline localization heavily interferes with the quantification of asymmetry. The technique is operator dependent, and the positioning of the patients has to be extremely precise. To avoid an altered localization of the midline, the patient's head must be centrally positioned, and the correct skull rotation has to be accurately checked to eliminate the possible artefacts both of linear and angular measurements [23]. Summarizing, a diagnostic error in the craniofacial asymmetry due to poor identification of implicated structures could lead to an erroneous treatment [30].

Our study population was composed of 47 adult subjects needing orthodontic treatment. The sample comprised 26 females and 21 males considering that no gender-associated difference in craniofacial asymmetry has been reported in the adult population [1]. Two types of landmarks were used to trace the midline of the cranial base: the ear rods of the craniostat (corresponding to the porion) and the spinosum foramina. These landmarks are relevant in defining the sagittal, transverse, and angular position of the condyles, are commonly used in the published literature, and are easy recognizable on the SMV radiographs [18].

Statistical analysis revealed the substantial equivalence and reliability of the two tracing methods for performing a cephalometric analysis in a representative population. This reliability results from the fact that the left/right discrepancy for Tracing 1 and 2 is not statistically significant (Table 2). Furthermore, the extent of asymmetry falls within a physiological range for both tracings, since an anthropometric value of 2-3 mm for the left/right cranial discrepancy, with respect to a midsagittal symmetry axis, is considered as the normal limit [31]. Trpkova et al. [1] underlined that there is no consensus concerning the right or left side prevalence in physiological asymmetries.

Our data indicate that some parameters may be more pronounced on the left or the right side, but that the side of prevalence of the same parameter can change if a different midline is established (Table 1). This result underlines the importance of midline localization for asymmetry considerations in orthodontic and surgical diagnosis, independently to the choice of 2D or 3D imaging, and could explain why some authors identified a right prevalence, whereas others identified a left prevalence.

Comparing the SR of the intra and extracondylar hemidistances, the statistical analysis showed a lower SR for Tracing 2 (0.99 ± 0.08 versus 1.06 ± 0.11 of Tracing 1 for intracondylar hemidistance and 0.98 ± 0.07 versus 1.04 ± 0.09 of Tracing 1 for extracondylar hemidistance; Table 2). This result has two different possible interpretations. The first one is that the finding is casual considering that a certain grade of asymmetry is always present and that TMJ internal derangements with bone modification (condylar hipo/hiperplasy, osteoarthrosis, osteonecrosis, osteoarthritis) could interest every subject independently to angle class. However, patients with a history of TMD have been excluded during the recruitment for the study reducing at the minimum this eventuality. The second possibility is that since the patients are Class 1 patients, they are the least likely candidates to develop TMJ anatomical and degenerative disorders and consequently the more symmetric subjects in the population [8]. In light of this, the MSP would be nearer than the MP to the ideal midsagittal axis, and this could explain why the SR is reduced for the Tracing 2.

The SR calculated using the MSP agrees with data obtained in a study on dry skulls performed by Marmary et al. [17] and demonstrates the high reliability of the spinosum foramina as references for midline localization.

The SMV images of the five control cases allowed us to verify the impact of successful therapy on the modification of the radiographic landmarks used to trace the symmetry axis. The distances between spinosum foramina and ear rods were identified on the radiograms to evaluate their changes over time. The repeatability of the cephalometric measurements before and after therapy was ensured by the same angle of incidence of the X-ray beam on the film thanks to the craniostat that maintains the position of the head unaltered. This projection allows also appreciation of the minimal variations in condylar dimensions and the relationship between mandibular condyles and basicranium [32].

In the three growing subjects, the millimetric increase of the absolute values between ear rods, corresponding to the distance between the external acoustic meatuses, is clearly evident. This change is the direct consequence of physiologic development: the advancing of the temporal bones and the new orientation of the glenoid cavities directly influence the position of the temporomandibular joint and acoustic meatus.

In all five cases, the distance between spinosum foramina remained unvaried (Table 3). Spinosum foramina belong to the central area of the basicranium that reaches adult dimensions in an early age and preserves its morphology throughout life [33]. Specifically, Sejrsen et al. [34] found that the central area of the external cranial base reaches its final extension at the age of 4-5 years: this area is delimited by the magnus foramen, by the stylomastoid foramina and by the spinosum foramina. Consequently, the growth of this area is very rapid until the age of 4-5 years and progressively decreases and eventually ceases after this age. For this reason, authors have suggested that the neurovascolar foramina can be used as references for the evaluation of the maxillomandibular complex.

As found by Moss and Salentijn [35], after this age, the dimensional stability of some structural aspects of this median area, among which the passage and the localization of neurovascular foramina, is not influenced by orofacial growth or orthognathodontic therapy. Conversely, major dimensional changes, both in the sagittal and transverse direction, take place during development in the contiguous anterior and lateral structures. This semilunar area includes also the glenoid cavities and the external acustic meatus, which are directly involved in the processes of bone remodelling during growth. It may also be hypothesized that masticatory function, facial trauma, and orthognathodontic therapy will influence the development of temporal bones but not the position of the neurovascular foramina.

Williamson et al. [18] demonstrated that the spinosum foramina show the lowest identification error in the submentovertex projection, whereas greater difficulty was encountered in identifying the lateral poles of the mandibular condyles. The authors also underlined that the use of ear rods for the determination of a reference plane may be suspect because of the tridimensional asymmetry associated with the external auditory meatus [36]. This asymmetry would provoke a head rotation during the positioning of the patient and a subsequent image distortion with a misinterpretation of the results [30]. Furthermore, the positioning of the ear rods in the acoustic external meatus could be influenced by the operator's skill and precision.

Consequently, if the analysis of the condylar asymmetries is performed in growing subjects, utilization of anatomic references such as the neurovascular foramina seems to guarantee a lower error than nonfixed references. This hypothesis needs to be confirmed by a larger cases series to test its statistical significance and understand whether this margin of error is clinically relevant for the precise quantification of craniofacial asymmetries.

One of the limitations of this retrospective analysis is that it lacks the comparison with 3D images although van Vlijmen et al. [37, 38] recommended to avoid the comparison between 3D tracings and conventional cephalometry in longitudinal research if there are only 2D records in the past.

## 5. Conclusions

Submentovertex radiograms can provide assistance in diagnosing condylar asymmetries and planning the most appropriate treatment; furthermore, the reliability of this examination allows assessment of the anatomic variations induced by the orthognathodontic or surgical therapy [2, 39]. A careful evaluation of physiologic condylar asymmetry is extremely important considering that if treatment on the craniomandibular complex does not respect this asymmetry, the risk of temporomandibular disorders can increase [8, 40].

The extent of the asymmetry can be quantified by using as a reference the midline, which should be as much as possible superimposable to the ideal midsagittal axis and not change during cranial development.

The results of this study validate our hypothesis indicating that the midline traced using the spinosum foramina as references more closely approximates the ideal midsagittal axis and represents the most reliable line to trace the coordinate system for identifying craniofacial asymmetry during cranial development on submentovertex radiograms.

## Figures and Tables

**Table 1:** Symmetry ratio of the variables analyzed.

Tracing 1	Mean	St Dev	Left/right symmetry ratio (SR)
Left condylar width (mm)	22.1	3.7	0.98 ± 0.17
Right condylar width (mm)	22.5	3.9	
Horizontal left condylar angle (grades)	21.5	7.9	0.98 ± 0.30
Horizontal right condylar angle (grades)	22.9	7.2	
LCoM-MP (mm)	50.1	4.9	1.06 ± 0.11
RCoM-MP (mm)	47.3	4.4	
LCoL-MP (mm)	71.2	6.8	1.04 ± 0.09
RCoL-MP (mm)	68.4	5.9	

Tracing 2	Mean	St Dev	Left/right symmetry ratio (SR)

Left condylar width (mm)	22.1	3.7	0.98 ± 0.17
Right condylar width (mm)	22.5	3.9	
Horizontal left condylar angle (grades)	20.9	7.5	0.98 ± 0.30
Horizontal right condylar angle (grades)	22.8	6.6	
LCoM-MSP (mm)	48.5	4.7	0.99 ± 0.08
RCoM-MSP (mm)	48.8	4.4	
LCoL-MSP (mm)	69.1	5.6	0.98 ± 0.07
RCoL-MSP (mm)	70.3	4.5	

**Table 2:** Statistical analysis comparing the differences in the symmetry ratio using Tracing 1 and Tracing 2.

Left/right symmetry ratio (SR)	Tracing 1	Tracing 2
Intracondylar hemidistance	LCoM-MP/RCoM-MP: 1.06 ± 0.11^a, c^	LcoM-MSP/RCoM-MSP: 0.99 ± 0.08^b, c^
Extracondylar hemidistance	LCoL-MP/RCoL-MP: 1.04 ± 0.09^a, d^	LCoL-MSP/RCoL-MSP: 0.98 ± 0.07^b, d^

^a^ *t*-test (C.I. 95%): *P* < 0.2 NS.

^b^ *t*-test (C.I. 95%): *P* < 0.9 NS.

^c^ *t*-test (C.I. 95%): *P* < 0.000.

^d^ *t*-test (C.I. 95%): *P* < 0.000.

**Table 3:** Scheme of the distances between the ear rods and the spinosum foramina for the five control cases.

control cases (years)	Ear rods distance (mm)	Spinosum foramina distance (mm)
BC 18	147.1 ± 0.1	72.6 ± 0.05
BC 21	148.3 ± 0.05	72.6 ± 0.05
GF 4	131.4 ± 0.2	64.1 ± 0.2
GF 5	134.0 ± 0.2	64.3 ± 0.1
SC 25	142.5 ± 0.1	80.7 ± 0.05
SC 28	142.6 ± 0.3	80.8 ± 0.1
PC 8	146.7 ± 0.05	77.1 ± 0.1
PC 11	150.2 ± 0.1	77.5 ± 0.2
KM 5	134.9 ± 0.2	69.6 ± 0.05
KM 8	142.3 ± 0.2	69.8 ± 0.05
